# Plants as the Extended Phenotype of Endophytes—The Actual Source of Bioactive Compounds

**DOI:** 10.3390/ijms241210096

**Published:** 2023-06-13

**Authors:** Natalia Rutkowska, Piotr Drożdżyński, Małgorzata Ryngajłło, Olga Marchut-Mikołajczyk

**Affiliations:** Institute of Molecular and Industrial Biotechnology, Lodz University of Technology, Stefanowskiego 2/22, 90-537 Lodz, Poland; piotr.drozdzynski@p.lodz.pl (P.D.); malgorzata.ryngajllo@p.lodz.pl (M.R.); olga.marchut-mikolajczyk@p.lodz.pl (O.M.-M.)

**Keywords:** endophytes, bioactive compounds, “extended phenotype”, habitat-adapted symbiosis, co-evolution

## Abstract

For thousands of years, plants have been used for their medicinal properties. The industrial production of plant-beneficial compounds is facing many drawbacks, such as seasonal dependence and troublesome extraction and purification processes, which have led to many species being on the edge of extinction. As the demand for compounds applicable to, e.g., cancer treatment, is still growing, there is a need to develop sustainable production processes. The industrial potential of the endophytic microorganisms residing within plant tissues is undeniable, as they are often able to produce, in vitro, similar to or even the same compounds as their hosts. The peculiar conditions of the endophytic lifestyle raise questions about the molecular background of the biosynthesis of these bioactive compounds in planta, and the actual producer, whether it is the plant itself or its residents. Extending this knowledge is crucial to overcoming the current limitations in the implementation of endophytes for larger-scale production. In this review, we focus on the possible routes of the synthesis of host-specific compounds in planta by their endophytes.

## 1. Introduction

In 1982, the evolutionary biologist Richard Dawkins introduced the concept of the extended phenotype in *The Extended Phenotype*, the sequel to his most famous book, *The Selfish Gene*. The main idea focuses on the extent to which genes can extend their effects beyond their possessors and the positive evolutionary consequences this has for them [1]. According to Dawkins, three forms of extended phenotypes can be differentiated. The first form applies to the single species involved and describes building architectural structures as a way of altering the environment to increase the chances of survival and reproduction (e.g., beaver dams [2]). In the second scenario, one organism can directly influence the behavior of another (some parasite–host interactions, for instance, in hairworm-infected crickets [3], ensuring their survival). The third form is related to the second, but the parasite acts indirectly on the host and uses mimicry to trick the host’s behavior for its own benefit (e.g., cuckoos tossing their eggs to other birds) [4]. Dawkins’s concept of an extended phenotype sparked a debate among evolutionary biologists [5,6,7], and since then, the definition has changed slightly and its meaning has expanded.

This phenomenon is also observed in plants. Lev-Jadun and Halpern (2019) described the concept of a double extended phenotype in relation to a plant’s capacity for creating sharp silica needles and inserting microbial pathogens into the tissues of herbivores [8]. This is a case of mutualism, in which the infection of the herbivore’s defenses directly benefits the plant, while simultaneously transferring pathogens into the tissues of the herbivore’s target, ensuring its fitness. Apart from this, as suggested by Hunter and revised by others, the plant–soil interaction is the primary example of a novel understanding of an extended phenotype, as plants and their associated microbiota can affect the soil composition by expressing their genes, thereby favoring the growth of one type of microbial or plant species over another [6,9,10,11]. Alternately, the plant itself can be considered to be the extended phenotype of the microorganisms inhabiting its tissues, whether as pathogens or non-causing apparent diseases, because they contribute to the overall phenotype of the plant by producing bioactive compounds themselves [12,13,14].

Plants constitute a main source of structurally diverse compounds with antioxidant [15], anticancer [16,17], anti-inflammatory [18,19], antimicrobial [20], neurological [21], and hepatoprotective properties [22]. Their usage in pharmacotherapy is as old as mankind itself, supported by written evidence such as the Sumerian clay slab from Nagpur (c. 3000 BC), the Chinese book on roots and grasses *Pen T’Sao* (c. 2500 BC), the Indian holy book *Vedas* (c. 1200 BC), *The Ebers Papyrus* (c. 1500 BC), and even references in *The Bible*, and Talmud and Homer’s *The Iliad* and *The Odyssey* [23]. Currently, plant-derived metabolites are extensively used in the food industry, cosmetics, and medicine. Furthermore, there is a growing demand for these bioactive compounds of a natural origin in the treatment of critical diseases such as cancer, diabetes, cardiovascular and respiratory diseases, and Alzheimer’s disease. It is estimated that the global market for botanical and plant-derived drugs will grow by 15.89 billion dollars from 2022 to 2026, at a CAGR (Compound Annual Growth Rate) of 7.25 percent, primarily due to the impact of COVID-19 on the rising interest in herbal medicines [24]. However, the conventional methods for extracting these compounds have many drawbacks, such as a seasonal dependence, very low yields of desired compounds, and troublesome extraction and purification procedures (often expensive and unsustainable with the use of chemicals), as well as difficulties in transportation due to geographical and political barriers (which can also spoil the quality and composition of the compounds) [25,26].

Additionally, the potential of plant-associated microorganisms should not be ignored. The most mysterious organisms among them are endophytes, which thrive inside plant tissues without causing apparent symptoms of disease [27]. They are often able to produce similar to or even identical compounds as their host plant [28,29]. Due to global warming, exploitation, and massive deforestation, we are witnessing the accelerated loss of biodiversity and the endangerment of numerous plant species. However, this phenomenon is resolvable in a number of ways. Endophytes can become a promising industrial source of these metabolites, due to the fact that they overcome the aforementioned limitations restricting plants and therefore prevent the extinction of these plants in numerous locations.

Based on the existing literature, the objective of this study was to explicate the actual source of the bioactive compounds present in plants, specially focusing on the extended phenotype of endophytes. The specific objectives of this review were to shed light on the plant benefits of habitat-adapted symbiosis with its endophytes, as well as their co-evolution and its impact on the molecular and physiological backgrounds of the synthesis of host-specific compounds in planta and ex planta.

## 2. Are Endophytes Crucial for Plant Existence?

The first person to describe “*Entophytae*”, German botanist Heinrich Friedrich Link, defined them as mostly parasitic fungi in 1809, and this standpoint did not change until M. Victor Galippe suggested in 1887 that both bacteria and fungi can migrate to plants from soil and have possible beneficial effects on host plants [30,31]. In the past, it was commonly acknowledged that healthy plants are generally free of microbes, a concept supported by other well-known scientists of the time, including Louis Pasteur and Auguste Fernbach [32]. Galippe’s ground-breaking and widely criticized hypothesis was the first, later confirmed by the works of Jorissen and Marcano [32,33].

Presently, there is no doubt that plants do not exist independently. The microorganisms associated with and/or interacting with plants can be divided into a small number of groups, mainly based on their location (rhizosphere, phyllosphere, and endosphere), but also depending on their level of connection with plant tissues and duration of habitat (Table 1).

According to the classic definition by Petrini (1991), endophytes are microorganisms that inhabit plant tissues at some point in their lifespan, without causing any apparent harm to the host plant [36]. There are almost as many proposed ways to classify these endophytes as there are their definitions, however, the most commonly used are the ”obligate” and ”facultative” subgroups. Obligate endophytes rely fully on their plant host to survive and are able to reproduce only inside plant tissues, transmitted mostly vertically through seeds, while facultative endophytes colonize plants from the rhizosphere or phyllosphere (horizontal transmission) when such opportunities arise [37]. Vertically transmitted endophytes can be co-cladogenetic, meaning that the evolution of the endophyte and host occur simultaneously and are inextricably linked; thus, regardless of the growth place and environmental conditions, the plant can host a specific taxonomy group to ensure the transmission of crucial symbionts [38,39]. The best-studied examples of such relationships are fungal endophytes from the *Epichloë* species of the *Poaceae* family of grasses [38,40]. In contrast, facultative endophytes can be recruited by the plant itself in response to environmental changes. Plant roots exude specific compounds (e.g., organic acids, amino acids, and flavonoids), which are recognized by rhizospheric microorganisms and trigger their movement towards roots [41,42]. For instance, Tian et al. (2021) reported that the presence of sucrose in root exudates promoted their colonization by *Bacillus subtilis*, which is a common bacterial species in soil with biocontrol activities [43]. Biofilm formation is the next crucial step for efficient colonization, followed by the penetration of the root surface and the colonization of the internal part of the plant [42]. Only some rhizospheric microorganisms are able to successfully colonize the plant and become endophytes: they are competent if they can beneficially modulate the plant physiology and maintain balance, therefore making them selectively favored or opportunistic if needed, occasionally profiting from the plant [34]. Sometimes, microorganisms enter plant tissues purely by chance, lacking the necessary traits for successful colonization, namely “passenger” endophytes. Both passenger and opportunistic endophytes are retained in the root cortex and are not allowed to transfer to the inner parts of the root; however, they still exhibit plant-growth-promoting traits [34,44,45].

Under normal growth conditions, endophytes can have neutral or beneficial effects on the host plant throughout their entire lifetime (so-called systemic endophytes); however, some endophytes can have detrimental effects that are beneficial to host plants under more extreme growth conditions or at different stages of a plant’s life cycle (non-systemic endophytes) [35]. For example, the fungus *Fusarium verticillioides* plays a dual role in maize, operating as both a harmful pathogen and a beneficial endophyte [46]. Pathogenicity experiments conducted on endophytic fungi isolated from a healthy *Axonopus compressus* plant revealed their capacity to infect both their natural hosts and non-hosts [47]. The equilibrium between these two states is determined not only by the genotype of the host, but also by the local abiotic stress factors that have a negative impact on the host fitness [37]. The endophytic fungus *Diploidia mutila*, which typically inhabits the harmless tissues of mature *Iriartea deltoidea* palm trees in Amazonian tropical forests, can be triggered by minor changes, such as a change in light intensity. When exposed to high light levels, fungus acts as a pathogen on seedlings, most likely as a result of the increased production of toxic hydrogen peroxide [48]. Nonetheless, switches can operate in either direction. Zhang et al. (2020) discovered that the small DNA mycovirus (SsHADV-1) causes the switch from pathogenic to endophytic in its host fungus *Sclerotinia sclerotiorum,* by downregulating its essential pathogenicity genes [49].

Small environmental changes can impair a plant’s morphological and physiological processes, thereby impeding its growth. This is especially true for crop plants, whose yields can be drastically reduced by drought, heat, and cold surges. Such events that cause abiotic stress are intensifying as a result of climate change and may have a significant impact on global production in future years, while the increasing human population is proportionally increasing the demand for food [50]. Clearly, plants have their own response mechanisms to various abiotic stresses by activating osmotic/oxidative stress signaling (osmolytes and abscisic acid production), LEA-type genes (Late Embryogenesis Abundant), and SOS (The Salt Overly Sensitive) pathways [51,52].

**Figure 1:**
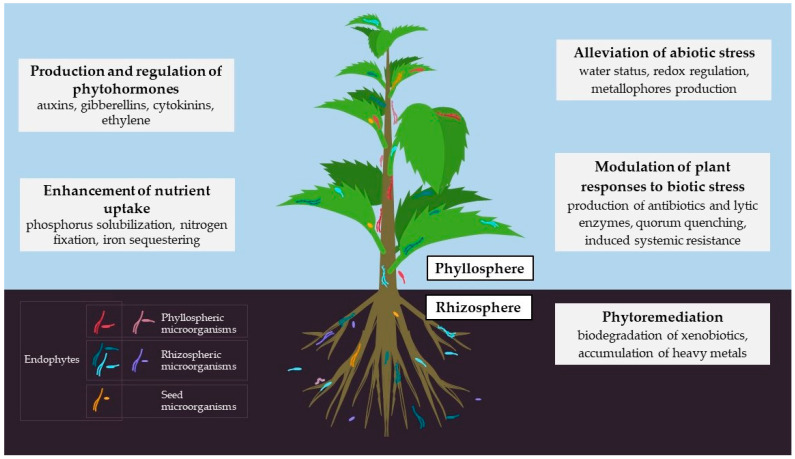
Plant-growth-promoting traits of endophytes.

Symbiosis with endophytes improves plant fitness in a number of ways, including enhancing their nutrient acquisition and providing protection against biotic and abiotic stresses. There is a classic example of a symbiotic relationship: the plant provides a steady flow of nutrients and protection, while the microbes thrive within and produce compounds that improve the plant’s fitness (Figure 1).

It is assumed that all plant tissues are colonized by endophytes (Bacteria, Fungi, and/or Archaea), so the existence of an endophyte-free plant in its natural habitat is highly unlikely [14,53,54,55]. Without endophytic support, plants, as sessile organisms, would be unable to adapt quickly and effectively to changing environmental conditions. The selection of plants to study the symbiotic relationships between plants and their associated microbiota based on their environmental setting is a great strategy for elucidating the significance of endophytes for the survival of plants, given that endophytes can display unique adaptations to extremophilic conditions. For instance, the fungus *Curvularia protuberata* inhibit the *Dichanthelium lanuginosum* plant tissues of those growing in Yellowstone National Park’s geothermal soil (20–50 °C). Redman et al. [54] studied endophyte-free and artificially endophyte-colonized plants, both in the laboratory and the field, and found that neither the host plant nor endophytic fungus could grow separately in temperatures above 40 °C. This led to the assumption that, in this case, plant–endophyte symbiosis is essential for heat tolerance, as endophytes can stimulate the plant stress response systems. In addition, only *C. protuberata* isolated from geothermal plants exhibited this ability, which was subsequently confirmed to be an indirect result of the presence of fungal RNA viruses in fungi [56,57]. The authors of the study called this phenomenon habitat-adapted symbiosis, after discovering a similar relationship between the endophytic fungus *Fusarium culmorum* and its host plant *Leymus mollis,* isolated from the beach coast of Puget Sound, Washington, in terms of salt tolerance [58]. In addition, inoculation with both of these fungi increased the heat and salt stress tolerance of commercially available rice varieties, indicating the industrial potential of endophytes isolated from extremophilic plants to mitigate the effects of climate change [59]. Similarly, Hosseyni Moghaddam et al. (2021) demonstrated that endophytic fungal species (*Periconia macrospinosa*, *Neocamarosporium chichastianum*, and *Neocamarosporium gogapense*) from desert-adapted plants have overall beneficial effects by enhancing the salinity and drought tolerance in cucumber and tomato plants under laboratory conditions [60]. During symbiosis under extremophilic conditions, fungi enhance the antioxidant activities in plants, preventing an excessive accumulation of reactive oxygen species (ROS); however, little is known about the endophyte-mediated regulation of the plant antioxidant enzyme system [60].

Apart from heat and salinity stress, plants mostly experience cold stress, as approximately 85% of the Earth’s biosphere is permanently exposed to temperatures below 5 °C [61]. Cold-adapted endophytes can thrive by adjusting their basic cellular processes to such conditions and expanding their abilities onto hosts. Araya et al. (2020) reported the presence of cold-tolerant hyper-ACC-degrading endophytic bacteria isolated from Antarctica’s two native vascular plants *Deschampsia antarctica* and *Colobanthus quitensis* [62]. Microorganisms with ACC (1-aminocyclopropane-1-carboxylate)-degrading abilities lower plant ethylene levels, which increase under stress conditions and inhibit further plant growth and development. All the endophytic bacteria survived freeze/thaw treatment (−20 °C for 24 h) and later grew on LB agar at 4 °C. They also exhibited significantly higher ice-recrystallization-inhibition (IRI) activities than phyllospheric, presumably producing antifreeze proteins (AFP) [62]. The cold is not the only stressful factor in Antarctica, as the stratospheric ozone layer over it is depleted. UV-B radiation (280–315 nm) impairs DNA and RNA, causes protein polymerization, increases cell membrane lipid peroxidation, and decreases photosynthesis, thereby resulting in delayed plant development and growth [63]. However, plants can mitigate the negative effects of UV-B by producing polyphenols, which can act as scavengers of reactive oxygen species (ROS) [64]. Barrera et al. (2020) investigated the fungal endophytes (*Penicillium chrysogenum*, *Penicillium brevicompactum*, *Alternaria* sp., *Phaeosphaeria* sp., and *Eupenicillium osmophilum*) isolated from *C*. *quitensis* for their ability to facilitate a high level of UV-B radiation [64]. The endophyte-inoculated *C. quitensis* plants mitigated the negative effects of UV-B radiation by decreasing their cell membrane lipid peroxidation and simultaneously increasing their flavonoid content after 48 h of treatment. Additionally, the expression of the plant genes involved in the mitigation of UV-R stress and the accumulation of flavonoids (*UVR8*, *HY5,* and *FLS* of the UVR8 pathway) was lower than that in the endophyte-free plants. All of the data indicate that endophytic fungi can facilitate the thriving of plants in extremophilic environments by activating certain molecular mechanisms e.g., the UVR8 pathway, some of which are yet to be revealed [64]. These observations are in accordance with Ramos et al. (2018), who also observed the beneficial modulation of the phytohormones in *C. quitensis* in the presence of fungal endophytes, suggesting that the plant immune system directly acts in the UV-B stress response [65]. A limited nutrient availability due to the slow rate of the decomposition of organic material also constitutes a restraining growth factor in cold environments, as well as the short-growing season itself. *Penicillium chrysogenum* and *Penicillium brevicompactum* isolated from *Colobanthus quitensis* and *Deschampsia antarctica,* respectively, exuded multiple hydrolytic and oxidative enzymes ex planta [66]. When reinoculated with their dominant endophyte, these plants showed a higher total biomass accumulation and nitrogen mineralization than endophyte-free plants. Thus, symbiosis with fungal endophytes directly supports the growth rate and nutrient acquisition of Antarctic plants [66]. These two fungal strains were also found to be industrially useful as inoculants of tomato and lettuce plants, not only facilitating salt stress and the overall survival rate of crops, but also increasing the total biomass and energy production of photosynthesis. An analysis of the plant gene expression during fungal symbiosis revealed that the overexpression of the *NHX1* gene is related to ion homeostasis, which resulted in the beneficial sequestering of Na^+^ in vacuoles [67]. Although many studies have focused on psychrotolerant endophytes, little is known about the direct mechanisms that stimulate host growth.

Some plants naturally grow in acidic soils and/or those contaminated with heavy metals such as Al, Cu, Pb, Zn, Sb, or As, which makes them potentially useful for phytoremediation. One of these, *Miscanthus sinensis*, is widely found in mine tailings in Asia. Haruma et al. (2018) managed to isolate the fungus *Chaetomium cupreum* from its roots, which showed high Fe- and Al-chelating activities. A re-inoculation of *C. cupreum* into sterile seedlings of *M. sinensis* significantly increased its growth in contaminated acidic soil and the dry weight of its above- and below-ground parts in comparison to endophyte-free seedlings. *C. cupreum* accumulated Al in its mycelia around the plant roots and in the epidermis, endodermis, and stele of these roots, thus reducing its toxicity [68]. The diazotrophic endophytic bacteria living in *M. sinensis* roots can also provide access to limited crucial nutrients in such environments, such as nitrogen, as they possess essential plant growth-promoting and metal(loid)-resistance genes [69]. The inhabitants of hyperaccumulator plants, common in mine tailings, often exhibit the ability to axenically remove heavy metals from liquid cultures ex planta [70,71,72]. In *Sedum plumbizincicola*, a re-inoculation of sterile seedlings with *Bacillus pumilus* E2S2 caused an increased soil mobility of Cd, Zn, and Pb (presumably due to siderophore production), plant biomass production, and accumulations of Cd and Zn in the host [71]. Taking all this into consideration, endophytes themselves can be potentially used as inoculants to extract metals from polluted soils under field conditions.

It is easy to see that far more studies have concentrated solely on endophytic fungi, and the effect of bacterial endophytes is underestimated, probably because fungi are considered to be easier to obtain from plants and more dominant. Tufail et al. (2022) conducted a global meta-analysis of 75 research papers from 2010 to 2021 to compare the effectiveness of endophytic bacteria and fungi in mitigating drought stress in crop plants. The endophytic bacteria improved plant productivity at a greater rate than the endophytic fungi (e.g., shoot and root dry mass, shoot and root length, photosynthetic rate, total chlorophyll content, proline content, leaf area, and abscisic acid content), regardless of the crop, experiment, or inoculation type [73]. Therefore, bacterial endophytes, which have been undervalued thus far, deserve more attention in future years.

Overall, the exploitation of beneficial endophytes, both fungal and bacterial, on crop plants to increase their yields at a larger industrial scale, constitutes an environmentally friendly strategy for satisfying the growing market demands, with the potential use of previously unfavorable places for cultivation.

## 3. The Actual Source of Plant Bioactive Molecules

In addition to plant growth-promoting metabolites synthesized by plant-associated microorganisms, endophytes are often capable of producing specific bioactive compounds that are similar or even identical to those of their hosts, especially medicinal plants [28,29,74,75]. Bioactive compounds are typically secondary metabolites of plant origin (mostly phenolics, alkaloids, and terpenoids, but also peptides and saccharides) with antimicrobial, antioxidant, anti-inflammatory, and anticancer properties [26,76].

Research on endophytes as the source of plant hosts’ bioactive compounds began in the 90s with the discovery of the fungal endophyte *Taxomyces andreanae*, which has the ability to synthesize paclitaxel (also known as Taxol), the first “billion dollar” anticancer drug, previously identified only in *Taxus* plants [77,78]. Since its isolation from trees is time-consuming, inefficient, and results in a low yield of taxol, this has sparked high hopes for an alternative resource and the development of a potential strategy for production directly from fungi [79]. To produce 1 kg of purified taxol, which is sufficient for treating 500 patients, 7000–10,000 kg of bark (750,000 trees) is needed [80]. So far, this production still relies on *Taxus* sp., threatening them with extinction; however, microbial alternatives have been extensively studied [81,82,83]. Similarly, camptothecin (CPT) is the other extensively studied compound, which is predominantly isolated from *Camptotheca accuminata* and *Nothapodytes nimmoniana* plants [84]. It acts as a DNA topoisomerase I inhibitor; thus, its derivatives, topotecan and irinotecan, are used in colon and ovarian cancer treatment [85]. The isolation of the endophytic fungus *Entrophospora infrequens* from *Nothapodytes foetida* was able to independently produce CPT in 2005 and was the first of many [86,87,88,89,90,91]. Notwithstanding this, these discoveries were followed by others, resulting in the acknowledgment of the endophytic potential of producing medicinally useful host-specific metabolites (Table 2), such as ginsenosides (*Panax ginseng*) [92,93,94], podophyllotoxin (*Podophyllum* species) [91,95,96,97,98,99], resveratrol (*Vitis vinifera*) [100,101,102,103,104], and vinca alkaloids (*Catharantus roseus*) [25,105,106,107].

A few of the studies listed in Table 2 include a direct comparison of plant and endophyte extracts [86,109,120,138,147], as this is not necessary for the identification of a compound. Since the bioactive molecules of medicinal plants are known, scientists look for their presence in microbial cultures using various chromatographic and spectrometric techniques, as described in detail by Mishra et al. (2022) [26].

It is now proven that endophytes contribute to the chemical composition of their host plant and stimulate the synthesis of host-specific bioactive compounds in planta [168,169]. For instance, Pandey et al. (2016) investigated the effect of fungal endophytes, *Curvularia* sp. CATDLF5 and *Choanephora infundibulifera* CATDLF6, on the metabolism of their host plant *Catharanthus roseus*. Re-inoculating sterile plants with these two endophytes significantly increased the vinca alkaloids content by 229–403%. In contrast to non-inoculated plants, the expressions of the key terpenoid indole alkaloid (TIA) pathway genes and other regulatory genes (*ORCA3* and *PRX1*) were upregulated in the inoculated plants, while the primary metabolic parameters remained unchanged [85]. Tiwari et al. (2013) also observed an increase in the vinca alkaloids content in the *C. roseus* plant after a re-inoculation with its endophytes, namely *Staphylococcus sciuri* and *Micrococcus* sp., as well as an increase in the plant growth [170]. In addition, the authors of these two aforementioned studies used various combinations of both endophytic bacteria and fungi to increase the *C. roseus* plant and vinca alkaloid content under field conditions during the winter and summer seasons, demonstrating the unquestionable viability of endophytic consortia as bioinoculants for promoting plant growth [171]. Re-inoculation with endophytes is not the only means of sustaining plant hosts. Biotic elicitors, such as the polysaccharide fraction from the endophytic fungus *Trichoderma atroviride*, stimulated the synthesis of tanshinones in *Salvia miltiorrhiza* by upregulating the involved plant genes [172]. It is evident that endophytes participate in the synthesis of bioactive compounds in planta, if not by manufacturing these compounds themselves, then by regulating the plant’s biosynthetic pathway.

Endophytes can produce similar or identical compounds to their plant host, examples of which are shown in Table 2. However, several obstacles prevent the commercial use of endophytes as alternative sources of these bioactive compounds. The yields are very low, far below industrially applicable levels, and successive subculturing decreases them even further, resulting in a general lack of production stability and making process optimization frequently impossible. Low yields and attenuation during the subculturing of taxol-producing endophytic fungi have prompted debate regarding their capacity for independent syntheses ex planta [173]. Heinig et al. (2013) suggested that endophytic fungi isolated from *Taxus* spp. plants could absorb taxanes into their cell walls and transfer them over for the first few subcultivations, resulting in a sharp decrease in the subsequent subcultures [173]. Numerous researchers have documented sudden reductions in the production of both endophytic fungi and bacteria. Devari et al. (2014) isolated the fungus *Alternaria alternata* from *Capsicum annum*, which is capable of producing capsaicin for up to three generations [116], whereas Shweta et al. (2013) observed attenuation after second subculturing in the camptothecin-producing bacteria of the *Bacillus* and *Lysinibacillus* species from *Miquelia dentata* [90]. However, as a general rule, the transmission of metabolites from host plants to endophytes appears dubious, as endophyte production is quite stable. In a study by El-Hawary et al. (2016), the attenuation of the solamargine production by *Aspergillus flavus* occurred after eleven generations [147], *Mucor fragilis* isolated and identified by Huang et al. (2014) produced podophyllotoxin for more than ten generations [88], and *Colletotrichum gloeosporioides* produced asiaticoside up to the seventh generation [112]. Since the conditions in planta are drastically different for isolated endophytes than those in their axenic cultures, it is also possible that their inability to maintain stable levels of secondary metabolites is due to a lack of metabolic crosstalk (precursors, transcription factors, and enzymes) between the endophyte, other plant-associated microorganisms, and the plant host, as well as gene silencing, the absence of host stimulation, or an unfavorable environment for that peculiar group of microorganisms [112,174]. In addition, the necessity for host stimulation has also been demonstrated. El-Elimat et al. (2014) observed a decrease in silymarin flavonolignans production following a subcultivation of the fungal endophyte *Aspergillus iizukae;* however, this production resumed on medium inoculated with *A. izzukae* spores grown on medium containing autoclaved leaves of the plant host *Silybum marianum*. After successive subculturing, the flavolignan production was again diminished [145]. Similar results were obtained by Li et al. (1998), in which the addition of *Torreya taxifolia* (host plant) extract reactivated the taxol production by fungus *Periconia* sp., indicating that the precursor or activator of the same stage of the biosynthetic pathway originated from the host plant. In addition, they discovered that few common activators of microbial metabolism have the ability to partially or even completely restore taxol production, while not being direct taxol precursors (benzoic acid, serinol, and gallic acid) [175]. In contrast, the findings of Gurudatt et al. (2010), who observed a decrease in the fourth generation of campothecin production by the endophytic fungus *Noithapodytes nimmoniana*, indicate that the addition of host tissue has no effect on reversing the production. However, the research team observed a decrease in the hyphal biomass despite an increase in the CPT concentration, leading them to hypothesize that CPT, as a secondary metabolite involved in defense, inhibits fungal proliferation [89]. In contrast, Vasanthakumari et al. (2015) were able to restore the CPT biosynthesis in the endophytic fungus *Botryospaheria rhodina* in vitro, which was significantly attenuated after the fourth subculturing, using two methods [176]. First, the attenuated fungi were inoculated onto the endophyte-free host plant, *Nothapodytes nimmoniana*, and then re-isolated, which resulted in an almost 3-fold higher yield (fungi isolated from the site of inoculation) than the average yield of the attenuated fungus. The authors also observed that passing the attenuated endophyte, *Phomopsis* sp. from *Miquelia dentata*, through non-host plants, but also producing CPT, resulted in reversal of this attenuation. The other method involved treatment with a DNA methyltransferase inhibitor (DNMT) and 5-azacytidine. In culture, 10 µM of 5-azacytidine increased the CPT production almost 2.5-fold [176]. Similarly, the addition of 1 µM of 5-azacytydine into the attenuated culture of the endophytic fungus *Diaporthe perseae* caused a significant increase in the colchicine production by 3.67-fold. The authors observed a gradual increase in the DNA methylation level on successive days of culturing, which was significantly reduced in the 5-azacytydine-treated culture [117]. Thus, endophytes that produce host-specific compounds require certain metabolic or molecular signaling, and in their absence, genes can be silenced by methylation. More detailed studies on the attenuation of production in both bacterial and fungal endophytes are needed to elucidate this process and decipher its molecular background.

## 4. Molecular Background of Host-Specific Compounds Synthesis

The molecular background of host-specific bioactive compound synthesis by endophytes remains a profound mystery. It is reasonable to assume that there is no general rule, as the relationship between plants and their associated microbiota can vary considerably. It is possible that the genomic pathway for the bioactive compound production of interest is dispersed throughout all the plant partners. For instance, Kusari et al. (2011) discovered that the biosynthesis pathway of CPT in an axenic culture of endophytic *Fusarium solani* cannot be entirely functional, as the fungus lacks a crucial enzyme (stricosidine synthase) responsible for converting CPT precursors into CPT [177]. The presence of stricosidine synthase in *Camptothecina acuminata* indicated the need for collaboration between the endophyte and the host. Still, CPT was produced in an axenic culture of fungus, and the reason for this is likely because the plant enzyme strictosidine synthase was carried over into the biomass of the isolated endophyte during its isolation in the right amount to produce CPT in the first two generations, with a significant drop from the third generation (what supports Heinig’s hypothesis) [173,177]. Interestingly, in the subsequent generations of the subculture, the CPT biosynthetic genes were altered and rendered inactive (the maintenance genes were unaffected), and no CPT intermediates were produced. An artificial inoculation of the endophytic fungus into its host plant, followed by re-isolation and axenic cultivation in vitro, did not restore the CPT synthesis, nor did the addition of a precursor provide satisfactory results. These findings provide clear evidence for a cross-species biosynthetic pathway occurring as a result of endophyte-plant symbiosis, as endophytes do not possess the enzyme analogous to stricosidine synthase, which would render its function useless [177]. Therefore, the selection of the appropriate unique plant genes involved in the biosynthesis of a selected compound as markers for screening its endophytes can be useful strategy, reducing screening time and efforts [178].

Another theory focuses on extrachromosomal DNA (ecDNA), such as organelles and plasmids, whose loss may be the genetic cause for sudden attenuations in both endophytic fungi and bacteria [179,180]. Extrachromosomal DNA elements are the primary agents of horizontal gene transfer; consequently, a host plant is able to transfer some of its genes onto its microbial locators [179,181]. Soujanya et al. (2017) reported CPT production by the endophytic bacteria from *Pyrenacanha volubilis*; however, this gradually decreased until the sixth subculture, where CPT was not detected. One of the studied strains, identified as *Bacillus subtilis*, acquired a 5 kbp plasmid resistant to ampicillin and did not produce CPR when cured. However, the CPT production was restored by plasmid transformation, both for the cured strain and attenuated strain [158]. Likely, the plasmid contained a crucial gene for the CPT biosynthesis pathway; however, only studying the whole gene sequence and gene expression patterns during CPT production by bacteria with plasmid could provide a solution to this matter.

The loss of endohyphal bacteria (EHB) during subculturing is another reason why the generation of bioactive compounds by fungal endophytes may be diminished [182,183]. The presence of bacteria within fungal hyphae was discovered nearly 100 years after the discovery of endophytes and described first as “bacterium-like organellas” in 1981 by MacDonald and Chander [184]. Since then, endohyphal bacteria have been studied rather poorly; however, it is known that they modulate fungal phenotypes and therefore have a direct impact on fungi–plant interactions [185,186,187]. They are often associated with the rhizospheric and endophytic fungi that affect seed germination and viability, as well as the plant microbiome [186,188]. Hoffman and Arnold (2010) described the diversity of the endohyphal bacteria in the living hyphae of the endophytic fungi associated with cupressaceous trees, using a combination of light and fluorescence microscopy [183]. Based on their 16S rRNA sequences, it was revealed that they represented *Proteobacteria* and *Firmicutes* phyla. No evidence for co-cladogenesis was observed and the transmission of both fungal endophytes and their bacterial symbionts was rather horizontal [183]. Later studies from the same research group on the previously isolated EHB *Luteibacter* sp. strain 9143 from the endophytic fungus *Pestalotiopsis* sp. strain 9143 revealed a significant enhancement of IAA production in vitro by the fungus in the presence of its endosymbiotic bacterium, thereby indirectly affecting the growth of the fungus host plant. Interestingly, bacteria do not produce IAA when grown axenically [185]. However, the results of RNA-sequencing experiments suggest that the *Luteibacter* strain may actually inhibit *Pestalotiopsis* growth and trigger its defense responses [189]. Thus, the subculturing of axenic fungal cultures (especially with the addition of antibiotics) may lead to the loss of endophytal bacteria, which could produce essential intermediate metabolites or be in possession of some part of the biosynthetic pathway, for example, on its own plasmid [179].

## 5. Co-Evolution of Plant and Its Endophytes

The presence of plant-associated microorganisms, including fungal endophytes, dates back to Devonian and Carboniferous periods (~300–420 million years ago). Surprisingly, the prehistoric connections between endophytic fungus and plants are structurally similar to modern ones [190]. Taking into consideration this evidence, it is indisputable that both vertically transferred endophytes and their plant hosts are affected by each other during long periods of co-existence and are able to successfully maintain their relationship (co-cladogenesis) [39]. This leads to horizontal (lateral) gene transfer events that, in certain severe situations, can result in the acquisition of genes. Horizontal gene transfer (HGT) is the non-genealogical transmission of genetic material between organisms living in the same environment [191,192]. Such transmissions can be accidental and infrequent, but if the gene obtained is beneficial for its new host, it can be promoted by selection (as antibiotic resistance genes). This is especially prevalent among bacteria, as it causes genome rearrangements, thus enhancing bacterial evolution [193]. Most HGT in bacteria occurs during transformation, transduction, and conjugation and typical mobile genetic elements (MGEs) include plasmids, transposons, integrons, and viral agents [194]. Recent years have also brought an interest in eukaryotic HGT as more and more whole genomes were sequenced, however, information on this matter is still scarce. HGTs from associated bacteria, fungi, and viruses could be crucial for the evolution of land plants, as the genes acquired by plants are associated with stress responses [195]. Although many endophyte researchers suspect the occurrence of such transfers from plants into microorganisms, which is why microorganisms can produce similar or even the same compounds, only a few such events have been examined enough to be considered likely [196].

The majority of studied HGT events, in terms of endophyte–plant relationships, are between plants’ endophytes. Taghvai et al. (2005) identified an endophytic bacteria *Burkholderia cepacia* VM1468 containing plasmid pTOM-Bu61, which is responsible for toluene degradation. An inoculation of poplar with the strain provided enhanced plant growth and reduced the amount of released toluene. Despite the fact that *B. cepacia* VM1468 did not manage to join the endogenous endophytic community successfully, its valuable plasmid was distributed across other bacteria, guaranteeing the community’s ability to degrade toluene [197]. Similarly, Wang et al. (2010) also observed the toluene-degrading endophyte *Burkholderia cepacia* strain FX2, whose plasmid contained the catechol 2,3-dioxygenase-encoding gene (from the pathway for the degradation of monocyclic aromatic compounds). The plasmid was transferred to the endogenous endophytic community of wheat and corn after its inoculation with the FX2 strain [198]. Both studies prove that HGT events mediated by plasmids containing valuable genes for plant fitness can happen even in a short time of a donor and its recipients coexisting. Transfer between plants and their endophytes is also possible. The root endophytes of *Ginkgo biloba*, participating in their host’s flavonoids and terpenoids syntheses, possess susceptibly homologous long terminal repeat retrotransposons (LTR-RT) to their host; thus, the presence of such MGEs prove that HGTs from plants to endophytes could happen at some point during their coexistence [199]. An in silico examination of whole genomes and the maximum similarity of single genes can also provide answers for such events from already established endophytic communities, therefore detecting HGT events that occurred a long time ago [200,201,202].

Horizontal gene transfer is not the only possible evolutionary route taken by endophytes. According to the xenohormesis hypothesis developed by Howitz and Sinclair (2008), under evolutionary selective pressure, plant consumers can sense and take plant-stress-induced chemical cues (mostly its secondary metabolites) as warning signals and begin to produce similar or even identical compounds via their indigenous homologous genes to activate their own defense system [203]. Endophytes are plant consumers, after all, so their peculiar lifestyle conditions, alongside their stress-mediating properties (e.g., producing growth-promoting, insecticidal, and antimicrobial compounds for pests and pathogen elimination), make them a perfect example of this hypothesis. Plants and their endophytes undergo the same selection pressure, since they live together in the same specific environment; therefore, this might lead to the development of similar or the same compounds. This concept, proposed as “trait-specific endophytic infallibility”, also elucidates why only a few endophytes from certain plants possess similar abilities, as this depends on the genome-specific features of both partners and is not accidental [124]. Endophytes exhibiting a certain phenotype possess an advantage that is favorable for the selective pressure of plant hosts and, over time, they may be “chosen” to be transferred vertically via seeds [204]. Such a process of the independent development of analogous pathways by two distinct organisms (plants and endophytes) is called convergent evolution [205].

The most elucidated example of convergent evolution is the biosynthesis of gibberellins by plant, fungi, and bacteria [206,207]. Gibberellins (GAs) are plant hormones that regulate plant growth by stimulating seed germination, floral and grain development, and sex expression [208]. The plant biosynthetic pathway was determined by studying the *Arabidopsis* genome [209]. The first steps of this pathway are similar in microorganisms too, as they involve the generation of GA_12_-aldehyde via the cyclization of GGDP (geranyl-geranyl diphosphate) and multiple oxidations of *ent*-kaurene and its derivatives via the MVA/MEP pathways [207]. In fungi, only the MVA pathway is involved and only one bifunctional enzyme (terpene cyclase) catalyzes the formation of *ent*-kaurene from GGDP, while, in higher plants and bacteria, two enzymes are needed (*ent*-copalyl-diphosphate synthase and *ent*-kaurene synthase). The subsequent step consists of conversions of GA_12_-aldehyde into other GAs, resulting in the synthesis of biologically active gibberellic acid 1, 3, and 4. The differences in this step between plant, fungi, and bacteria are significant because of their enzymatic machinery, therefore indicating the independent evolution of their gibberellins pathways [206,207,209,210]. These are described in detail by Salazar Cerezo et al. (2018) and Hedden (2020) [207,211]. However, it is hard to define clearly the origin of host-specific compound synthesis in endophytes, and not many attempts have been made. So far, the biosynthetic pathway of taxol production by endophytes is the most studied. Zhang et al. (2009) compared the sequences of the *dbat* gene (10-deacetylbccatin-III-10-O-acetyl transferase) involved in taxol biosynthesis from *Cladosporium cladosporiodes* and its host plant *Taxus media*, which resulted in a 99% similarity, indicating the possibility of HGTs in acquisition of that gene by endophytes [212]. However, Xiong et al.’s (2013) research shows that the other important genes for taxol synthesis—*ts* (taxadiene synthase) and *bapt* (C-13 phenylpropanoid side chain-CoA acetyl transferase)—in the endophytic fungi from *Taxus media* (*Guignardia mangiferae*, *Fusarium proliferatum*, and *Colletotrichum gloeosporioides*) have low levels of similarity with the genes from the host, suggesting an independent evolution in both partners [213]. Findings from Yang and others (2014), who compared 13 candidate genes of paclitaxel biosynthesis in the endophytic fungus *Penicillium aurantiogriseum* with its host *Corylus avellana* and the *Taxus baccata* genomes, support this theory [214]. On the contrary, Sah et al. (2017) isolated the endophytic fungal strain *Lasiodiploidia theobromae* from the *Piper nigrum* plant (definitely a non-*Taxus* species), which was able to independently produce taxol [215]. The predicted amino sequence of the fungal *dbat* gene was found to be homologous with the taxol-producing plant *Taxus cuspidata* and other fungal *dbat* genes. This suggests the possibility of the occurrence of HGTs, however, as approximately 19 enzymes are needed in taxol biosynthesis, it is not possible to transfer all of them through HGTs. Apparently, taxol production could have evolved independently in fungal endophytes and their plant hosts, and some of the 19 enzymes—f.e. coded by the *dbat* gene, as discovered by Sah et al. (2017)—may have been transferred via HGTs [215]. Miao et al. (2018) examined the expressions of the genes involved in the taxol production of the endophytic fungus *Cladosporium cladosporioides* MD2 after subculturing with a partial attenuation of the taxol production. The authors managed to identify potential partial taxol biosynthetic pathways and suggested that *C. cladosporioides* MD2 uses different pathways than *Taxus* to produce the intermediate 10-DAB compound (10-deacetyl-2-debenzoylbaccatin III). The later steps also differed from the *Taxus* pathway—no homologous enzyme genes, *dbat*, *bapt*, or *dbtnbt* (3′-N-debenzoyltaxol N-benzoyltransferase), were found in the fungal transcriptome. This may be direct reason for the attenuation of taxol production during subculturing, as other enzymes could be responsible for that stage, but with no apparent nucleotide sequence identities of *Taxus* enzymes, which were therefore not detected and annotated [216]. The study by Qiao et al. (2020) also used a transcriptome analysis to investigate the molecular background of taxol biosynthesis by endophytic fungi; here, using *Aspergillus aculeatinus* Tax-6, they proved that endophytes possess genes involved in the isoprenoids synthesis pathway that are similar to the *Taxus* sp. plants’ genes. The authors also provided answers for the low yields of taxol production, as they did not observe the expressions of most downstream genes involved in the hydroxylation, acetyl group transfer, and benzoyl acylation of the side chains on C13 on the taxol backbone, which can explain the low yield of production. However, they identified a few steps contributing to taxol biosynthesis, such as the upregulation of the genes involved in the cell cycle, which led to a higher biomass production, and genes related to glycine metabolism [217]. Thus, it is possible that these endophytes produce taxol on their own, since they possess most of the crucial upstream genes (gained by horizontal gene transfer or developed independently), but lack the key specific downstream genes, which restrains them from achieving higher yields.

## 6. Future Prospects

To the best of our knowledge, none of the identified endophytic isolates with the potential to produce host-specific compounds have been industrialized. First, endophytic bacteria have been studied less than endophytic fungi, due to the fact that fungi are simpler to obtain and more abundant; therefore, bacteria are at a disadvantage from the start. The primary challenge for both endophytic bacteria and fungi is the reduction in their secondary metabolite production upon repeated subculturing under axenic monoculture conditions, due to the differences in their synthetic pathways in planta and ex planta—the absence of selective pressure, precursors and/or transcription factors, host stimulus/selective pressure, and the necessary host genes. The entire process underlying the biosynthesis of host-specific metabolites by endophytes is still a mystery, despite the revolution in “omics” technologies, which has shortened the time required for analyses over the last two decades. In addition, although endophytes with the ability to produce the same compounds as plants are not rare, it should be made clear that only a small number of isolated microorganisms from a single plant are capable of doing so. To discover such gems, extensive screening and more effective isolation procedures are required. The best target to search for host-specific compound synthesis is seed endophytes, as they are primarily transmitted vertically from generation to generation and thus co-exist the longest with their plant host. They could develop parallel pathways for their synthesis to ensure a better fitness for their plant, and by extension, their own health.

Endophytes should not be regarded as “standard” microorganisms when studying the production of host-specific metabolites. As their living conditions are unique, cultivation methods imitating the conditions present in planta (e.g., maintaining salinity or drought stress, heavy metal stress, different temperatures, pH or oxygen level, co-culturing, or adding a plant’s host extract) may activate the biosynthetic genes and induce the synthesis of a plant’s host-specific compounds.

It is essential to elucidate the genetic foundation of such biosynthesis. The horizontal gene transfer phenomenon is the most enticing explanation for host-specific compound production by endophytes; consequently, it should be thoroughly investigated to presume that this is the case. It could be accomplished by searching the genome in silico for suspicious-looking elements (by using e.g., Alien_hunter, GIPSy) or by using a simpler degenerate PCR technique if there are putative genes. Importantly, studies on this topic should include not only the selected endophyte and its host plant, but also the co-existing microorganisms, as genetic determinants can be distributed among all partners. In addition, it is essential to compare non-endophytic and endophytic strains of the same genus, in order to identify and comprehend the differences that contribute to the biosynthesis of host-specific compounds by endophytes and provide evidence for gene development events. Complex screening for the HSC production by endophytes and a subsequent determination of its genetic background are clues for the optimization of production, development of efficient production methods (possibly in other model microorganisms), and, consequently, future industrial applications, not to mention answers to numerous questions regarding endophytes’ biology.

## Figures and Tables

**Table 1:** Types of plant-associated microorganisms [34,35].

Microorganisms	Living Conditions/Specifics
Rhizospheric	soil in close proximity to roots and its exudates
Rhizoplanic	surface of plant roots
Phyllospheric (=epiphytes)	surface of aerial parts of a plant
Endophytic (=endophytes)	inside plant tissues without causing any apparent harm to plant host
Obligate	living inside a plant during their entire lifespan
Facultative	optionally living inside of a plant
Systemic (true)	strictly symbiotic, non-pathogenic at any stage of its lifespan
Non-systemic (transient)	live asymptomatically within plant for a part of their lifespan, can turn into pathogen when plant host is stressed or resource-limited
Competent	successfully colonize plant, can alter its physiology and be selectively favored
Opportunistic	occasionally enter a plant and benefit from its internal environment
Passenger	enter a plant accidently in the absence of selective forces for efficient root colonization

**Table 2:** Endophytes producing host-specific bioactive compounds of medicinal use ex planta.

Compound	Properties	Endophyte	Plant Host	Amount Produced by Endophyte Ex Planta	Reference
**Fungi**					
Aconitine	Analgesic, anti-inflammatory, anti-tumor	*Cladosporium cladosporioides* XJ-A C03	*Aconitum leucostomum*	236.4 µg/g ^1^	[108]
Alternariol	Cytotoxic	*Alternaria* sp.	*Polygonum senegalense*	ND	[109]
Andrographolide	Anti-inflammatory, antineoplastic, anti-platelet aggregation	*Colletotrichum* sp. (AP-4, AP-12)	*Andrographis paniculata*	30.089 ± 0.992 mg/g ^1^ (AP-4), 28.617 ± 0.641 mg/g ^1^ (AP-12)	[110]
Asarone	Anticonvulsant, GABA modulator	*Penicillium pinophilum*	*Alloteropsis cimicina*	ND	[111]
Asiaticoside	Antioxidant, anti-inflammatory, antirheumatic	*Colletotrichum gloeosporioides*	*Centella asiactica*	62.29 ± 3.36 µg/100 mL ^2^	[112]
Azadirachtin	Hepatoprotective, insecticide	*Eupenicillium parvum*	*Azadirachta indica*	0.4 µg/100 g ^1^; 43 µg/L ^2^	[113]
Cajaninstilbene acid	Analgesic, antioxidant, anti-inflammatory, hypoglycemic, neuroprotective	*Fusarium proliferatum*	*Cajanus cajan*	100.5 ± 9.4 µg/g ^1^; 504.8 ± 20.1 µg/mL ^2^	[114]
Camptothecin	Anticancer (inhibition of DNA topoisomerase I), potential antineoplastic agent	*Fusarium solani* (MTCC 9667, 9668)	*Apodytes dimidiata*	37 µg/100 g ^1^; 53 µg/100 g ^1^	[86]
		*Fusarium solani*	*Camptotheca acuminata*	150 ± 20 µg/L ^2^	[88]
		*Alternaria alstroemeriae* (NCIM1408)	*Nothapodytes nimmoniana*	426.7 ± 33.6 µg/g ^1^	[115]
		*Alternaria burnsii* (NCIM1409)		403.3 ± 41.6 µg/g ^1^	
		*Fusarium* sp.		2.17 µg/100 mg ^1^	[89]
Capsaicin	Analgesic	*Alternaria alternata*	*Capsicum annuum*	8.30 µg/L ^2^	[116]
Colchicine	Anti-gout, anti-inflammatory	*Diaporthe perseae*	*Glorosa superba*	55.25 uµg/g ^1^	[117]
Deoxypodophyllotoxin	Precursor for podophyllotoxin, anticancer	*Aspergillus fumigatus*	*Juniperus communis*	4 ± 2 µg/100 g ^1^; 3 ± 2 µg/L ^2^	[118]
Dendrobine	Anti-cataract, anti-influenza A virus, and anti-tumor, promising therapeutical effects on Alzheimer’s disease	*Trichoderma longibrachiatum* MD33	*Dendrobium nobile*	ND	[119]
Digoxin	Anti-arrhythmia drug, cardiotonic drug	Unidentified	*Digitalis lanata*	ND	[120]
Diosgenin	Anticancer, antiatherogenic, antineoplastic, and antiviral agent	*Aspergillus flavus*	*Dioscorea zingiberensis*	ND	[121]
		*Curvularia lunata*			
		*Fusarium* sp.			
		*Paecilomyces* sp.	*Paris podophylla*	ND	[122]
Emodin	Antioxidant, anti-inflammatory, antimicrobial, hepatoprotective, precursor to hypericin	*Epicoccum nigrum*	*Hypericum perforatum*	87.7 µg/mL ^3^	[123]
		*Thielavia subthermophilia*		113 ± 1 µg/100 g ^1^	[124]
Forskolin	Antihypertensive, anti-HIV, platelet aggregation inhibitor	*Rhizactonia bataticola*	*Coleus forskoholii*	0.5 mg ^2^	[125]
Gentiopicrin	Antifungal, antihistamine, anti-inflammatory	Unidentified (QJ18)	*Gentiana macrophylla*	ND	[126]
Ginsenoside Rg3	Antioxidant, antidiabetic, antineoplastic agent, apoptosis inducer	*Chaetomium* sp.	*Panax ginseng*	5.60 ± 1.17 mg/g ^1^	[92]
Gymnemagenin	Antidiabetic, antiviral	*Penicillium oxalicum*	*Gymnema sylvestre*	ND	[127]
Huperzine A	Neuroprotective, treatment of Alzheimer’s disease	*Acremonium* sp.	*Huperzia serrata*	8.32 µg/L ^2^	[128]
		*Shiraia* sp. Slf14		327.8 µg/L ^2^	[129]
Hypericin	Antidepressant, anti-inflammatory, antineoplastic, immunostimulating	*Epicoccum nigrum*	*Hypericum perforatum*	117.1 µg/mL ^3^	[123]
		*Thielavia subthermophilia*		35 ± 2 µg/100 g ^1^	[124,130]
Imperialine-3β-D-glucoside	Anti-tumor	*Fusarium redolens* 6WBY3	*Fritillaria unibracteata*	18.8 µg/L ^2^	[131]
Loline alkaloids	Insecticidal	*Neotyphodium uncinatum*	*Lolium pratense*	700 mg/L ^2^	[132]
Kaempferol	Antioxidant, antibacterial	*Mucor fragilis*	*Sinopodophyllum hexandrum*	ND	[96]
Myrtucommulones	Anti-oxidant, anti-inflammatory, anti-tumor	*Neofusicoccum australe*	*Myrtus communis*	0.2 mg/L ^2^	[133]
Nigranoic acid	Anti-neoplastic agent, HIV-1 reverse transcriptase inhibitor	*Umbelopsis dimorphia*	*Kadsura angustifolia*	ND	[134]
Peiminine	Anticancer, anti-inflammatory	*Fusarium* sp.	*Fritillaria unibracteata*	0.021 mg/L ^2^; 0.0054 mg/g ^1^	[135]
Peimisine	Anticancer, anti-inflammatory, angiotensin-converting enzyme (ACE) inhibitor	*Fusarium* sp.	*Fritillaria unibracteata*	0.09 mg/L^2^; 0.0023 mg/g ^1^	[135]
		*Fusarium redolens* 6WBY3		16.0 µg/L ^2^	[131]
Piperine	Antioxidant, anti-inflammatory, antimycobacterial, insecticidial, increasing bioavailability of drugs	*Colletotrichum gloeosporioides*	*Piper nigrum*	ND	[136]
		*Periconia* sp.	*Piper longum*	750 mg ^3^	[137]
Podophyllotoxin (PTOX)	Antimitotic, anti-tumor, precursor for anticancer drugs, e.g., etoposide and teniposide	*Fusarium* sp. (WB5121)	*Dysosma versipellis*	277 µg/g ^1^	[97]
		*Fusarium oxysporum*	*Juniper recurva*	28 µg/g ^1^	[98]
		*Fusarium solani*	*Podophyllum hexandrum*	29.16 ± 0.57 µg/g ^1^	[95]
		*Phialocephaa fortinii*	*Podophyllum peltatum*	189 µg/L ^2^	[99]
		*Trametes hirsuta*	*Podophyllum hexandrum*	31 µg/g ^1^	[91]
		*Mucor fragilis*	*Sinopodophyllum hexandrum*	49.3 µg/g ^1^	[96]
Quercetin monoglycosides	Antioxidant, stimulating bacterial enzymatic activity	*Nigrospora oryzae*	*Loranthus micranthus*	ND	[138]
Quinidine	Antiarrhythmic, antimalarial	*Diaporthe* spp. (CLS-3)	*Cinchona ledgeriana*	82.5 µg/L ^2^	[139]
Resveratrol	Antioxidant, anti-inflammatory, antimutagen, antiviral, phytoestrogenic	*Alternaria* sp. MG1	*Vitis vinifera*	353 µg/L ^2^	[103]
		*Arcopilus aureus*		89.1 µg/mL ^2^	[101]
		*Botryosphaeria* sp.		37.3 µg/mL ^2^	
		*Nigrospora* sp.		25.2 µg/mL ^2^	
		*Aspergillus stellifer* AB4		300 µg/L ^2^	[104]
		*Fusarium equiseti*		52.3 µg/mL ^2^	[100]
		*Quambalaria cyanescens*		40 mg/L ^2^	[102]
Rhein	Anticancer, anti-inflammatory, antimicrobial, hemostatic	*Fusarium solani*	*Rheum palmatum*	5.672 mg/L ^2^	[140]
Saikosaponin d	Anti-inflammatory, anti-tumor, immunomodulatory	*Fusarium acuminatum*	*Bupleurum scorzonerifolium*	2.40 µg/mL ^2^	[141]
		*Fusarium oxysporum*		2.17 µg/mL ^2^	
Salidroside	Adaptogenic, antioxidant, antidepressant, anti-inflammatory, neuroprotective	*Phialocephala fortinii* (Rac56)	*Rhodiola angusta*	2.339 ± 0.1093 mg/mL ^2^	[142]
Salvianolic acid C	Anticancer, antioxidant, treatment of cardiovascular and cerebrovascular diseases	*Phoma glomerata* D14	*Salvia miliorrhiza*	0.054 µg/mL ^2^; 47.67 ± 0.04 µg/g ^3^	[143]
Saponin	Anti-inflammatory, antimicrobial, anti-ulcer, haemolytic, hepatoprotective	*Fusarium* sp. (Pg27)	*Panax ginseng*	0.181 mg/mL ^2^	[144]
Silybin A and B, isosilybin A	Anti-tumor, hepatoprotective	*Aspergillus iizukae*	*Silybum marianum*	0.13–0.22 µg/g ^3^	[145]
Sipeimine	Anti-tumor, antitussive	Unidentified (Fu7)	*Fritillaria ussuriensis*	ND	[146]
Solamargine	Antidiabetogenic, cytotoxic	*Aspergillus flavus*	*Solanum nigrum*	250–300 µg/L^2^	[147]
Tanshinone I and IIA	Anti-inflammatory, anticoronaviral, anticancer	*Trichoderma atroviride* D16	*Salvia miliorrhiza*	1.119 ± 0.008 µg/g ^1^ (I); 3.049 ± 0.001 µg/g ^1^ (IIA)	[148]
Taxol (paclitaxel)	Chemotherapy drug	*Annulohypoxylon* sp. MUS1	*Taxus wallichiana*	282.05 µg/L ^2^	[149]
		*Aspergillus fumigatus*	*Taxus* sp.	1.60 g/L ^2^	[150]
		*Seimatoantlerium nepalense*	*Taxus wallichiana*	ND	[151]
		*Seimatoantlerium tepuisense*	*Venezuelan guyana*	250–350 ng/L ^2^	[152]
		*Taxomyces andreanae*	*Taxus brevifolia*	24–50 ng/L ^2^	[77]
Tyrosol	Antioxidant, cardioprotective	*Phialocephala fortinii* (Rac56)	*Rhodiola angusta*	2.002 ± 0.0009 mg/mL ^2^	[142]
Vinblastine	Antineoplastic, anti-tumor	*Curvularia verruculosa*	*Catharanthus roseus*	182 µg/L ^2^	[105]
		*Fusarium oxysporum*		76 µg/L ^2^	[25]
		*Fusarium solani*		ND	[107]
		*Talaromyces radicus*		70 µg/L ^2^	[153]
Vincamine	Antihypertensive, vasodilator	*Geomyces* sp.	*Nerium indicum*	1.279 mg/L ^2^	[154]
		Unidentified (Vm-J2)	*Vinca minor*	0.1 mg/L ^2^	[155]
Vincristine	Chemotherapy drug	*Eutypella* sp.	*Catharanthus roseus*	53 ± 5.0 µg/L ^2^	[106]
		*Fusarium oxysporum*		67 µg/L ^2^	[25]
		*Fusarium solani*		ND	[107]
Viniferin	Anti-inflammatory, anticancer, anti-angiogenic, antimicrobial, anthelminthic	*Aspergillus stellifer* AB4	*Vitis vinifera*	324 µg/L ^2^	[104]
Withanolide	Antioxidant, anti-inflammatory, antistress, cardioprotective, neuroprotective	*Taleromyces pinophilus*	*Withania somnifera*	360 mg/L ^2^	[156]
**Bacteria**					
Achillin	Anti-inflammatory, antihypertensive, vasorelaxant	*Microbacterium maritypicum*	*Ephedra foliata*	ND	[157]
Berberine	Analgesic, anti-inflammatory, antimicrobial, hypolipidemic	*Kytococcus schroeteri*	*Ephedra foliata*	ND	[157]
		*Paenibacillus polymyxa*			
Camptothecin	Anticancer (inhibition of DNA topoisomerase I), potential antineoplastic agent	*Bacillus cereus* ChST	*Miquelia dentata*	1.177 µg/mL ^2^	[90]
		*Bacillus subtilis* PXJ-5		1.554 µg/mL ^2^	
		*Bacillus subtilis*	*Pyrenacantha angustifolia*	0.18 µg/mL ^2^	[158]
		*Kytococcus schroeteri*	*Ephedra foliata*	ND	[157]
Daunorubicin	Antibiotic, antineoplastic, anticancer	*Paenibacillus polymyxa*	*Ephedra foliata*	ND	[157]
Dendrobine analogs	Anti-cataract, and anti-influenza A virus, anti-tumor, promising therapeutical effects for Alzheimer’s disease	*Pseudomonas proteogens* CHA0	*Dendrobium* sp.	ND	[159]
Diosgenin	Anticancer, antiatherogenic, antineoplastic, and antiviral agent	*Bacillus* sp.	*Trigonella foenum-graceum*	527.83 µg/L ^2^	[160]
		*Bacillus cereus*		156.33 µg/L ^2^	
Galanthamine	Treatment of Alzheimer’s disease	*Paenibacillus lautus*	*Leucojum aestivum*	37.51 µg/g ^1^	[161]
		*Burkholderia graminis*	*Narcissus tazetta*	37.8 µg/L ^2^	[162]
		*Bacillus thuringiensis*		61.0 µg/L ^2^	
Ginsenoside Rg3	Antioxidant, antidiabetic, antineoplastic agent, apoptosis inducer	*Burkholderia* sp.	*Panax ginseng*	ND	[93]
		*Agrobacterium* sp. (PDA-2)		62.20 mg/L ^2^	[94]
Harmine	Anti-inflammatory, anti-tumor, hallucinogen	*Microbacterium maritypicum*	*Ephedra foliata*	ND	[157]
Ligustrazine	Anti-inflammatory, nootropic	*Bacillus subtilis*	*Ligusticum chuanxiong*	1.0268 mg/L ^2^	[163]
Lycorine	Anticancer	*Paenibacillus lautus*	*Leucojum aestivum*	37.51 µg/g ^1^	[163]
Maytansine	Antimicrobial, antineoplastic	Unidentified	*Putterlickia retrospinosa*	ND	[164]
			*Putterlickia verrucosa*		
Myricetin	Antioxidant, anti-inflammatory, antithrombotic, antidiabetic, neuroprotective	*Microbacterium maritypicum*	*Ephedra foliata*	ND	[157]
Oxylipins	Antibiotic	*Bacillus* sp.	*Alternanthera brasiliana*	ND	[165]
Taxol (paclitaxel)	Chemotherapy drug	*Kitasatospora* sp.	*Taxus baccata*	ND	[166]
Sanguinarine	Antibiotic, anticancer	*Paenibacillus polymyxa*	*Ephedra foliata*	ND	[157]
Vindoline	Antimitotic, precursor for vinblastine	*Microbacterium* sp.	*Catharanthus roseus*	82 ug/L ^2^	[167]

ND—no data; ^1^—dry weight of mycelia; ^2^—in culture medium; ^3^—in extract.

## Data Availability

Not applicable.
